# On the Connexion between Morbid Physical and Religious Phenomena

**Published:** 1854-10-01

**Authors:** J. F. Denham


### ON THE CONNEXION BETWEEN MORBID PHYSICAL AND RELIGIOUS PHENOMENA.

No. I. of a Series.

BY THE REV. J. F. DENHAM, M.A., F.R.S., &c.

It is a received medical opinion that all bodily diseases affect the mind, that is, cause a change to some degree and extent or other of its perceptions, feelings, &c., and the mode whereby this change is produced is well ascertained, and level to every one's apprehension. Thus, the brain is the material organ or instrument of the mind, and upon its healthy state the mind depends for its correct action. It is itself liable to diseases, it sympathizes with any diseased part through its nervous connexion with it. It is extremely sensitive to the abnormal qualities, velocity, &c. of the blood produced by many diseases, and to any chemical and mechanical affections of the nerves, especially of the stomach and other digestive organs. It would seem highly reasonable to expect that the changes thus produced by bodily causes on the mind would extend to its religious phenomena; and this probability is often alluded to in the writings of the most learned and pious divines, who also advert to the necessity of taking it into consideration in judging of spiritual maladies, and of paying practical regard to it in the treatment of such maladies.

The following observations upon the subject are the results of an attention paid to it throughout a long and diversified clerical experience, aided by such an acquaintance with the best medical works as was compatible with my more directly professional duties and studies. My plan of observing has been to ascertain the nature of the disease in the sick cases to which I have been called to minister, if possible, from the medical attendant; and to make memoranda of the spiritual phenomena by which it was accompanied in its various stages. In now, however, communicating the results of my observations and reading, no *recognizable* use will be made of any particular cases that have come under my notice.

In order to secure to my observations, as far as I am able, the benefits of precision and method, and which are peculiarly needed in the treatment of a subject supposed by many persons to involve something beyond mere *physical* causes, I beg to premise those definitions and principles which appear to me to be essential to the correct and useful examination of it.

First, in order to form any practical idea of the diseased phenomena of body or of mind, it is necessary to form such an idea of their healthy phenomena. "Pour connaitre homme malade, il faut connaitre homme sain."*

By healthy physical phenomena may be understood such as attend an adult body free from malformation and from structural and functional disease: and by healthy religious phenomena such as are displayed by the mind united to such a body, possessing the ordinary degree of ability, and which has received the usual amount of intellectual culture along with good moral training, and a correct and intelligent religious education; and the religious phenomena which may be expected in such an instance of "a sound mind in a sound body," would be characterized by that *medium* in which propriety of all kinds so usually consists; or by the *balance being preserved* between its intellectual, moral, and religious powers—especially between the judgment and the emotions—and particularly by the absence of unintelligent and disproportionate excitement; and evinced chiefly in the following qualities: neither insensible nor excitable, neither sceptical nor superstitious: docile but not credulous: humility without abjectness, cheerfulness without elation, seriousness without despondency, devotion without enthusiasm; inclination to moral and religious improvement without spiritual pride or ambition: no intense attention to itself or to the minds of others; more impressed with the necessity of moral and religious duty than of an acquaintance with theories and abstractions. It is difficult of course, if not impossible, to give a perfect definition of healthy and diseased phenomena either of body or of mind; perhaps they are best referred to our instinctive ideal of them; or the reader may possibly find some exemplification of them in the instances of the more happy, useful, and respectable portion of his acquaintance; but the preceding description may sufficiently serve the purposes of a standard. Accordingly all physical and religious phenomena may be considered as morbid which deviate from that standard, and in proportion as they deviate from it.

2. By the connexion between morbid physical and religious phenomena is to be understood, not that every kind and degree of the former is attended by some kind or degree of the latter, but that no kind of the latter ever exists except in union with some kind of the former, more or less clearly marked.

3. I submit that the chief rules of philosophising, Regulæ Philosophandi, as established by Sir Isaac Newton, and which Hartley contends ought to be adhered to with equal rigour in our attempts to solve the phenomena of the mind,† may be legitimately as well as advantageously applied to our subject. (1.) That no more causes of phenomena are to be admitted than what are real and sufficient to explain appearances: (2.) That phenomena of the same sort are to be accounted for by the same cause.

With regard to the application of the first of these rules to our subject it may be remarked that "the reality" of morbid physical phenomena is unquestionable, and that their "sufficiency to explain" morbid religious phenomena is evinced by the following facts: that the latter are not greater in extent or variety than those morbid mental phenomena which are attributed and traced by medical writers to bodily diseases; that both these classes of mental phenomena equally begin, increase, decline, and terminate with bodily diseases; that such religious phenomena never exist without morbid mental phenomena of a more general character; and that when the nature of the disease and the

† Hartley on Man, ch. i. p. 1.

sex, age, temperament, and other circumstances of the patient are duly estimated, a tolerably accurate expectation may be formed of the religious phenomena his mind will exhibit. With regard to the application of the second of these rules to our subject it may be remarked that the religious phenomena we denominate as morbid are evidently "of the same sort" as diseased mental phenomena in general, with the sole and unimportant distinction that they involve religious ideas, feelings, &c., and therefore are to be referred to the same cause.

The application of the foregoing rules to the subject, liberates it from the question of Satanic or demoniacal agency, and exempts us from the necessity of inquiring whether, and how far, the inspired writers do, or do not, really teach, and as part of the revelation they were commissioned to divulge, that either Satan or the demons cause any alteration of the phenomena of the human mind, either indirectly by the infliction of those bodily diseases from which morbid mental phenomena including the religious naturally result, or by direct agency, or by taking advantage of the debility produced naturally by bodily disease on the mind to exert upon it their supernatural power. It might, however, seem worthy of remark, that the religious phenomena in question yield, in the majority of instances, to medical remedies along with those other morbid mental phenomena of a more general nature with which they are always more or less associated. But would any one maintain that infernal agency may be dislodged from the mind by medical prescriptions?

We conceive ourselves then left at liberty to consider the relation of morbid religious phenomena to secondary and proximate and consequently to physical causes, and we are authorized by the Divine Founder of the Gospel to speak of secondary and physical causes, in his large admission of them when he says, "the earth bringeth forth fruit of herself"* αὐτομάτη ἡ γῆ &c. It may also best comport with the views of those who consider the mind to be an absolutely pure intelligence, apart from all possible material qualities, to conceive of it as being itself incapable of infirmity, and to regard all mental infirmity, as it is called, as originating in the infirm state of the physical organ of the mind, that to use the words of Christ, "the spirit is indeed willing, but the flesh is weak."† To those also who think that the physical origin of abnormal religious phenomena is attended with sufficient probability, it will appear, perhaps, further recommended by the considerations that it is both simpler and less gloomy than that of infernal agency, and therefore more in accordance with the appearances of nature and the scriptural representations of the benevolence of Deity, and of his perfect supremacy over all the works of his hands. It also directs the attention of the spiritual physician, and indeed of all persons, to the restoration and the care of bodily health as the readiest means of removing or preventing morbid religious phenomena. The experience, indeed, of every one even for a single day, of the connexion between his bodily states and his ideas, will be sufficient, if attended to, to convince him of the practical value of our subject. It is not too much to say that ideas are developed by physical states of a nature correspondent to those states, if indeed they be not also originated by them, and that those ideas which are in the first instance addressed to the mind, affect the body, according to its healthy or diseased susceptibility, and, as thereby modified, react upon the mind.

As already intimated, the connexion between morbid physical and religious phenomena, is adverted to in the writings of the most eminent and exemplary divines, and especially those of them who had turned their attention to medical studies, or, who like Abp. Secker, Dr. Barrow, and many others, had originally received a medical education. From these writings admissions may be produced, of the modifying influence of organization and of physical causes, not only upon the religious and intellectual, but even the moral phenomena of human nature, commensurate with the largest demands made for them by modern pathologists. Thus Bishop Beveridge remarks, "Atheistic thoughts spring up in the fountain of the soul *only* when mudded with fleshly pleasures."* Dr. Barrow observes, "Credulity may spring from an airy complexion; suspiciousness hath its *birth* from an earthly temper of the body."† Reserving other quotations for the particular occasions for them which will be afforded by the future examination of specific classes of morbid religious phenomena, I shall now subjoin the following *general* confirmation of the foregoing definitions, principles, &c., derived from the works of Abp. Sharp. "We consist of two parts, a soul and a body, which though they are distinct substances are yet by the wonderful power of God so closely united that they do strongly affect one another. Though it be our minds, or our spirits, or our souls, properly, that can be said to think, or to reflect, or to perceive, or to remember, or to hope, or to fear, or to enjoy, and the like; yet *all* these operations are influenced by, and do receive a kind of tincture as I may say from that state and condition and plight, the body is in. For it is plain, by manifold experience, that our souls in this world cannot act at all without the help and ministry of the purer parts of our bodily substance; which purer parts, let them consist in what they will, the soul makes use of as her instruments in *all* her intellectual operations, and as these are well or ill disposed, so will *all* the acts of our minds proceed accordingly. The changes and various dispositions that we feel in ourselves proceed not so much from the soul, for the habits and dispositions of that are often, for all these varieties, the very same, but rather they are in a great measure, if not wholly, to be attributed to the variety of tempers that the body is subject to, which the soul cannot many times either prevent or alter. *Irresolution* and *doubtfulness* about the goodness or badness of actions, as fearing that if we act this way we sin, if we act the other way we sin likewise, do often render the minds of well-meaning persons very uneasy, even sometimes in such instances as another man, and he an honest man too, would find no difficulty at all in. It may, and doth sometimes happen, that this perplexity and scrupulosity about actions doth proceed from distemper and indisposition of body; and when it does so, it is a spice of religious melancholy, and which is a dejection of mind occasioned from the temperament, or most commonly from the distemperature of the body, accompanied with unreasonable frights or fears about our spiritual condition. There is none in mankind can live a more uncomfortable life than they also do who are often thus dejected and under such sad fears and perplexities as sometimes to think themselves the most miserable wretches that breathe. Nay, even at their death, when they stand in need of comfort most, yet now and then it happens that they cannot rid themselves of those frightful and dismal apprehensions .... As for the devil, they neither give him opportunity, nor is he, I hope, ordinarily permitted to be so busy about them, as they are apt to imagine. No; I take it, that the principal causes and foundation of *all* their troubles lie in the ill habits of their bodies: the animal spirits, which the soul makes use of as her instruments in the performance of *all* her rational operations, are vitiated and disordered by fumes arising from hypochondriac affections, and *that* gives the first occasion to the disorder of their minds. That what I say is true, appears in this, namely, that those who are constantly and habitually thus troubled in mind, are known, by a great many symptoms, not only to be persons of a melancholy complexion, but also to be highly under the power of hypochondriac melancholy; and those that are not frequently under these troubles, but only sometimes, may observe of themselves that these troubles have usually come upon them, either upon some heavy cross and affliction that has befallen them, or some great sickness

* Private Thoughts, Art XI. † Sermon IX.

of which they were not well recovered, or some other natural cause that hath put their bodies into some weakness or indisposition, and when that has been removed, they have been as well in their minds as before .... Two things are necessary to be done for the cure or removal of religious melancholy, namely, that the persons afflicted with it do take care of their bodies, that they be put into a better state of health and vigour, and freed from all hypochondriac fumes that do oppress them; and that they endeavour to get their minds truly informed about those matters of religion, from which their disease doth, as I may say, take a handle to vex and disturb them. To speak my thoughts freely, I must needs say that in many of these cases the physician's part is every whit as necessary, if not more, than that of the divine; for if the bodily indisposition was removed, most of the fears, and frights, and disturbances that happen upon a religious account would vanish of themselves; whereas, while the root of the disease, I mean that ill ferment of the blood and spirits, remains in the body, the most comfortable discourses that can be made to them about their spiritual condition, though to the bystanders that hear them they appear never so wise and rational, will often have little effect on them; or if they do give them some present ease and satisfaction, yet in a little time their troubles and fears return again, and are as impetuous as they were before. I know that many of these will not give credit to what I now say. A man, for instance, that is troubled with horrid blasphemous thoughts will think it strange that you should advise him, for the cure of sin, to make use of physic and exercise, and such other methods as are prescribed to valetudinary persons for the recovery of their health: why, saith he, I am well enough in body, I eat, I drink, I sleep; all my disease is in my mind: I would be rid of these wicked thoughts that do continually haunt and torment me, and what can physic or exercise contribute to that? I have need of a spiritual physician. And so far, indeed, he is in the right. A spiritual physician may do him some service, and give him some comfort by convincing him, if he be capable of it, that these thoughts of his, how wicked and blasphemous soever they are, shall do him no harm so long as he doth not consent unto them. But this is all he can do. He cannot, I doubt, put him into the way of getting rid of these thoughts, which is the main thing he desires; for that cannot be done but by the alteration of the state of his body, from the ill-disposition of which *all* these thoughts do arise. But now the man being ignorant of all this, and having no idea how his body should thus affect his soul, as to the making him think after this or that manner, which yet it certainly doth, cannot readily entertain any advices that are given him with relation to that, though yet he will find upon trial that it is from hence *only* that his cure can be perfected. . . . Why may we not ascribe all that inequality we find in our affections towards God and spiritual things *wholly* to the inequality of the temper of our bodies? Without doubt this is generally the cause of it. As long as we have these bodies about us, the best of men must expect these ebbs and flows of affection to the service of God, and that even when they are in good health; and, therefore, much more if it should be their misfortune to have their animal spirits depraved by hypochondriacal affections. . . . No man that has seriously attended to the working of his own mind but will experience that he hath often had very odd and extravagant thoughts come into his head on a sudden, and those vigorously enough impressed, without any occasion that he can give account of, where there has not been the least reason to suspect that the devil had any hand in infusing them: but, as there is great reason to believe they did purely and solely arise from the present temper and motion of his animal spirits, which accordingly as they move regularly or irregularly, more briskly or more slowly, have a power of exciting in the soul thoughts and fancies of a differing nature. And hence come all the extravagances of dreams, the odd flights and recoveries of those that are in feverish distempers, and *likewise* the strange conceits and fancies of melancholy and hypochondriacal persons. It is not all persons that do complain of these wicked and blasphemous thoughts and other extravagant fancies, nor all good persons that are thus haunted, but chiefly those that are of a melancholy constitution—those of the devout sex, women, are more thus affected than the other sex. These that I speak of are grievously disturbed with odd, unreasonable—nay, sometimes impious phantasies, which are suggested to their minds, they do not know how, nor upon what occasion; but the more they strive against them still the more impetuously do they come into their heads; and then especially when they set themselves to the more solemn exercise of religion, and endeavour to be more than ordinarily devout, at these times, to be sure, they shall be most grievously tormented with them. What now shall we say to these things? I verily believe that, for the most part, they are wholly to be ascribed to the distemperature of our bodies, occasioned by hypochondriac vapours, or hysteric passions, or ill affections of our natural humours, and that the devil hath no hand in them. But if, after all this, any man will say that those thoughts do not take their rise wholly from bodily distempers, but that also the devil hath a hand in them—namely, thus far, that he takes advantages of those disorders in our humour, and by means thereof doth rather excite these thoughts in us, or impress them more vehemently upon us, which is, indeed, the common opinion of divines,—I say, if any one thinks this to be a better account of the matter, he may, for all me, enjoy his own sentiments; for I account religious melancholy, properly so called, to be as perfect a disease, and in some cases as incurable, as some other diseases incident to human bodies: but in most cases it is capable of cure; and in all cases it may receive great comfort, and relief, and abatement. *It concerns all these persons to look after their bodies, for upon the cure and health of them the cure and health of the mind doth, in a manner, all in all depend.*"—*Casuistical Sermons*, I.—V., Vol. III. London, 1716.

